# Early cancer detection by SERS spectroscopy and machine learning

**DOI:** 10.1038/s41377-023-01271-7

**Published:** 2023-09-15

**Authors:** Lingyan Shi, Yajuan Li, Zhi Li

**Affiliations:** Shu Chien-Gene Lay Department of Bioengineering, UC San Diego, La Jolla, CA 92093 USA

**Keywords:** Optical techniques, Optics and photonics

## Abstract

A new approach for early detection of multiple cancers is presented by integrating SERS spectroscopy of serum molecular fingerprints and machine learning.

Cancer is a major public health problem and the second leading cause of death worldwide, with 10 million deaths in 2020^1^. Early detection of cancer or precancerous change allows for early intervention and can improve survival rates. However, early detection of many cancers, such as esophageal and ovarian cancers, is still poor, which are often diagnosed at advanced stages^2^. This is more likely due to multiple remaining challenges, such as finding and validating biomarkers for multicancer types, and technologies applied for cancer detection^2^.

Current approaches for cancer screening and detection include blood tests, histopathology tests, imaging tests (for example, mammogram for breast cancer and computerized tomography for lung cancer), and genetic tests. Compared to histopathology and imaging tests, blood tests, which rely on the analysis of specific analytes and tumor biomarkers such as tumor DNA/protein and circulating tumor cells^3^, are noninvasive and more efficient. However, due to the challenge of validating many potential biomarkers for different cancer types, only a few biomarkers have been validated and used in clinic^4^.

Since the past decade, Raman and infrared spectroscopies have emerged for cancer diagnosis by detecting molecular vibrational fingerprints^5^. Studies utilized infrared spectroscopy and detected breast, bladder, prostate, and lung cancers via molecular fingerprints in blood plasma and serum^6,7^. Compared with infrared spectroscopy, various types of Raman spectroscopy are more commonly used for cancer detection. Among these, surface-enhanced Raman spectroscopy (SERS) measurement on blood samples is most frequently used in early cancer detection due to its high sensitivity and specificity^8–12^. Nevertheless, these studies were constrained by their utilization of a very limited set of biomarkers, rendering them unsuitable for detecting multiple types of cancer. Additionally, they lacked efficient data analysis methods. In contrast, the application of machine learning techniques has proven valuable in classifying serum biomarkers to detect cancer and other diseases using omics data^13^. However, machine learning requires a large independent dataset, while in the previous study only 10 out of a thousand dimensions could be selected for the machine learning algorithm^13^.

In their article in *Light*^14^, Shilian Dong and colleagues presented a method for early cancer detection that uses label-free SERS spectroscopy and machine learning for cancer screening (SERS-AICS) of serum biomarkers. The workflow of SERS-AICS is shown in Fig. 1. The authors analyzed 382 healthy controls and 1582 patients across five cancer types, including lung, colorectal, hepatic, gastric, and esophageal cancers. The SERS-AICS method successfully distinguished cancers from healthy controls with a high overall accuracy (95.81%), sensitivity (95.40%), and specificity (95.87%). Moreover, the method was able to separate precancerous samples from other diseases. This method has important implications for early cancer detection.

**Fig. 1:**
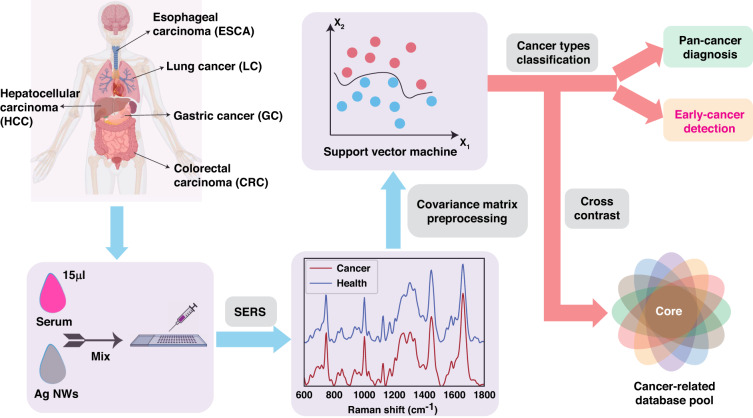
Schematic of SERS-AICS workflow. Serum samples were used for label-free SERS spectrum detection enhanced by silver (Ag) nanowires. After the dimensionality reduction of covariance matrix of high-dimensional serum spectral data, cancer-related dimension database of bond level can be obtained. Support vector machine algorithm was then used for classification. Figure is modified from ref. ^14^

In this study, only 15 µl of serum sample each for control and five cancers was used for SERS pan-cancer screening, in which the best SERS enhancement was achieved by using 70–120 nm-diameter silver nanowires. After Raman spectra of a large number of serum samples were collected, Raman feature dimension was trimmed by using covariance matrices, and the dataset of different cancers was classified by support vertical machine (SVM). In the SVM model, 50 intervals were chosen within the Raman fingerprint range (600–1800 cm^−1^). Thus, this method can screen up to 50 Raman feature dimensions, compared to 10 in the previous study. The SVM model could precisely distinguish subtle differences between spectral data and differentiate all five cancers from control with a high accuracy (ranging 94.10–98.25%), sensitivity (91.84–98.75%), and specificity (90.63–98.57%).

Another challenge of early cancer detection is the discrimination of cancer from other diseases of the same organs. By using the same strategy on serum from four types of diseases (lung, colorectal, gastric, hepatic) and corresponding cancers, gastric and hepatic (diseases/cancer) groups were separated with high accuracy (>92.3%), sensitivity (100% and 85.7%) and specificity (85.7 and 100%); lung and colorectal groups could also be separated except with a slightly lower specificity (77.78%) and sensitivity (66.67%), respectively. Moreover, the SVM classification is able to analyze a large amount of data (~2000 cases). This large sample pool allows for more reliable spectral data across different cancers. Hence, shared Raman peaks by all five types of cancer, combinations of any types of cancer, or precancerous and diseased samples can be characterized and potentially used as common features for differentiating cancers.

This study combines SERS analysis of serum for cancer screening with machine learning techniques for cancer classification. This innovative approach offers a highly sensitive and selective method for distinguishing between multiple types of cancers, not only from normal samples but also from non-cancerous diseased samples. By utilizing a large database model training and validation, spectra of all molecules present in the serum can be comprehensively collected and analyzed, enabling multiple accurate discrimination of subtle differences among various cancers and controls. The newly proposed approach offers a new venue for identifying these cancers at their earliest stages. If this innovative method can replicate its outstanding performance on prospectively collected samples in a meticulously double-blinded manner, it can be implemented as a screening test in the clinics and ultimately save lives.
